# Fully hydrogenated vegetable oil-based non-dairy creamer intake impairs athletic performance in mice: serum metabolomics and intestinal microbiota analyses

**DOI:** 10.3389/fnut.2025.1657205

**Published:** 2026-01-06

**Authors:** Shi Qi Xu, Wenchao Gong, Jing Li, Wenjie Qin, De Xin Dang, Yanming Yu, Dong-Hwa Chung

**Affiliations:** 1 Institute of Physical Education, Hubei University of Arts and Science, Xiangyang, China; 2 Department of Endocrinology, Xiangyang Central Hospital, Affiliated Hospital of Hubei University of Arts and Science, Hubei University of Arts and Science, Xiangyang, China; 3 The First Affiliated Hospital of Guangzhou Medical University, Guangzhou Medical University, Guangzhou, China; 4 School of Basic Medicine, Hubei University of Arts and Science, Xiangyang, China; 5 Department of Sports Convergence Technology, Sangmyung University, Seoul, Republic of Korea

**Keywords:** additive, gut microbiota, inflammation, metabolomics, muscle function, oxidative stress, saturated fatty acids, sports food

## Abstract

**Introduction:**

Fully hydrogenated vegetable oil-based non-dairy creamer (FHVO-NDC) is produced through complete hydrogenation of vegetable oil, resulting in a trans fat-free product commonly incorporated into sports foods and performance-oriented beverages. Because diet plays a crucial role in shaping athletic performance, understanding the potential physiological effects of FHVO-NDC is essential for athletes and fitness enthusiasts. However, the combined impact of FHVO-NDC on muscle physiology, serum metabolomic alterations, and gut microbiota has not been evaluated. Therefore, the objective of this study was to address this knowledge gap using a controlled mouse model.

**Methods:**

Experimental animals were assigned to two groups: a control group receiving regular water and a treatment group receiving water supplemented with FHVO-NDC. Multiple physiological and biochemical parameters were monitored, including body condition (*n* = 8), feeding behavior (*n* = 4), athletic performance (*n* = 8), myofiber characteristics (*n* = 6), muscle fatigue-related biochemical indicators (*n* = 6), serum metabolite profiles (*n* = 5), and intestinal microbiota composition (*n* = 5). Data were analyzed using one-way ANOVA to determine statistical significance.

**Results:**

FHVO-NDC intake did not alter body condition or feeding behavior. However, it resulted in a measurable decline in athletic performance. This reduction was accompanied by decreased myofiber diameter (*p* < 0.001), reduced muscle glycogen content (*p* < 0.001), and lower levels of key enzymes associated with muscle fatigue (*p* ≤ 0.001). Additionally, FHVO-NDC consumption led to a significant increase in the abundance of the genus *Alistipes* (*p* = 0.007) and elevated levels of serum 2-fluoronicotinic acid (*p* = 0.022), both of which have been linked to impaired muscle function and metabolic dysregulation.

**Discussion:**

Collectively, these findings suggest that FHVO-NDC consumption may exert detrimental effects on muscle physiology and athletic performance in the mouse model. The observed alterations in muscle traits, serum metabolites, and gut microbiota highlight potential biological mechanisms through which FHVO-NDC could negatively influence exercise capacity, warranting further investigation regarding its implications for athletes and physically active individuals.

## Highlights

- FHVO-NDC intake impaired athletic performance in mice.
- Mice consuming FHVO-NDC showed marked reductions in myofiber diameter, muscle glycogen content, and the levels of key enzymes associated with muscle fatigue.
- FHVO-NDC consumption resulted in an increased abundance of intestinal bacteria commonly associated with adverse physiological effects.
- Serum metabolomics analysis demonstrated that FHVO-NDC intake promoted the accumulation of metabolites linked to impaired muscle function.

## 1 Introduction

Sports nutrition supplements, high-protein energy bars, and meal replacement protein-based solid beverages are widely consumed by athletes and fitness enthusiasts to restore performance after exercise and to optimize training outcomes (1). To improve the palatability of these processed foods and beverages, non-dairy creamer is frequently incorporated to enhance taste, texture, and overall sensory appeal (2, 3). Specifically, the addition of non-dairy creamer provides a thick and milk fat-like mouthfeel (3). In addition, its convenient storage, stable quality, and low production cost have driven its rapid global expansion across the food industry. The major component in non-dairy creamer responsible for generating this creamy mouthfeel is hydrogenated vegetable oil (4).

However, hydrogenation of vegetable oils introduces potential health concerns. Partial hydrogenation produces trans fats, which are well known to exert adverse metabolic and cardiovascular consequences (5). Full hydrogenation eliminates trans fats, producing fully hydrogenated vegetable oil-based non-dairy creamer (FHVO-NDC), which is primarily composed of saturated fats (6). Growing evidence has suggested that excessive intake of saturated fats may increase the risk of metabolic syndrome (7, 8) and promote chronic low-grade inflammation (9). Importantly, metabolic syndrome has been linked to reductions in muscle mass and strength across multiple studies (10, 11), while chronic inflammation has been associated with impaired athletic performance and delayed recovery (12). Furthermore, consumption of non-dairy creamer has been associated with obesity risk, gut microbiota dysbiosis, oxidative stress, lipid metabolic disturbances, and increased cardiovascular burden (13, 14). These findings raise the possibility that, although functional foods and performance-oriented beverages provide nutritional benefits to athletes and fitness enthusiasts, the inclusion of non-dairy creamer may diminish or counteract these advantages (15).

**Figure 1:**
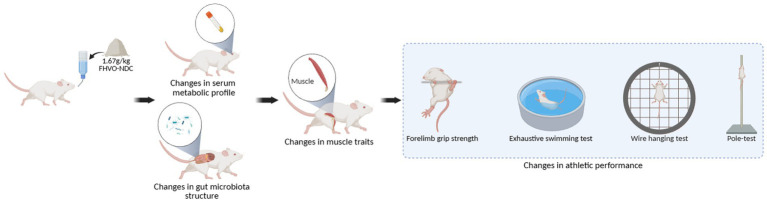
Schematic diagram illustrating the mechanism by which fully hydrogenated vegetable oil-based non-dairy creamer intake impairs athletic performance in mice.

Despite the widespread use of FHVO-NDC in sports-related foods, the combined effects of this ingredient on muscle physiology, metabolomic profiles, and gut microbiota have not been examined in a controlled experimental model. To address this research gap, the present study proposes a hypothesis: intake of FHVO-NDC impairs muscle traits by altering intestinal microbiota composition and serum metabolite profiles, ultimately leading to reduced athletic performance (Figure 1).

Mice were selected as the experimental model because the core physiological and molecular systems underlying exercise capacity, such as muscle contraction mechanics, mitochondrial energy metabolism, and neuromuscular regulation, are highly conserved between mice and humans (16). Moreover, murine models allow precise control of diet, environment, and activity, enabling the identification of relationships that are difficult to isolate in human populations due to confounding factors (17). Therefore, mice represent an appropriate *in vivo* system for evaluating the physiological impacts of exogenous food additives, providing mechanistic insights that are relevant to human athletic performance (18, 19).

Given that athletic performance is closely influenced by body condition, feeding behavior, myofiber characteristics, biochemical fatigue markers, serum metabolites, and gut microbial composition, the objective of this study was to investigate how FHVO-NDC intake affects these parameters using a controlled mouse model. The anticipated findings will contribute to a deeper understanding of how FHVO-NDC consumption may influence exercise capacity and recovery, offering meaningful dietary implications for athletes, fitness enthusiasts, and the sports nutrition industry.

## 2 Materials and methods

### 2.1 Experimental design

The experimental design was developed with reference to previous studies that evaluated the health effects of food additives using mouse model (20–22). A total of sixteen male C57BL/6 J mice (6 weeks old; mean initial body weight: 22.19 ± 7.22 g) with comparable body size and overall health status were used in this study. All mice were assessed through handheld inspection, including examination of the skin and fur, eyes, respiratory activity, tremors, convulsions, and general behavior (23, 24). Mice that did not meet these criteria were excluded. Because all animals shared the same genetic background and health condition, the experimental cohort was considered homogeneous (25). This study began in mid-September 2024 and ended in late November 2024, spanning a total of 70 days. Thus, the mice aged from 6 to 16 weeks during the experiment. Based on developmental timeline translations using neuroimaging-based tractography (26), mice aged 6–16 weeks correspond to humans aged approximately 15–25 years, a period characterized by rapid muscle development (27). The mice were randomly and evenly assigned to two groups: the control group (CON; *n* = 8) and the treatment group (TRT; *n* = 8). The only difference between the two groups was the composition of the drinking water. Mice in the CON group received regular water, whereas those in the TRT group received water supplemented with FHVO-NDC (Nestlé, Dongguan, China) at a concentration of 0.167%. FHVO-NDC consisted of glucose syrup, hydrogenated vegetable oil, stabilizers (340ii, 331ii, 452i), emulsifiers (471 and 472e), anti-caking agent 551, and casein. Per 100 g, FHVO-NDC provided 2,244 kJ of energy, 2 g of protein, 34 g of fat, 56 g of carbohydrates, and 490 mg of sodium.

The dosage of FHVO-NDC was determined based on estimated human daily intake. A standard commercial beverage contains 500 mL of water with 2% FHVO-NDC, equivalent to 10 g per day for an adult consuming 500 mL. Assuming an average adult body weight of 60 kg, this corresponds to 0.167 g/kg. Following the dose-conversion method described by Jin et al. (28), low and high mouse doses are typically set at 5- and 20-fold the recommended human amount. Therefore, a 10-fold multiple was chosen, resulting in a final dosage of 1.67 g/kg for mice.

To ensure that mice in the TRT group received an FHVO-NDC intake equivalent to 0.167%, the concentration of FHVO-NDC in drinking water was calculated using the following formula (29, 30):


Daily intake concentration=daily water intake×FHVO−NDCconcentrationbody weight

where daily intake concentration is expressed in mg/kg/day; daily water intake in mL/day; FHVO-NDC concentration in mg/mL; and body weight in kg.

In this study, daily water intake and body weight were measured biweekly. Accordingly, the target daily intake concentration was fixed at 0.167%, and the required FHVO-NDC content in the drinking water was recalculated and adjusted every 2 weeks. Before administration, FHVO-NDC was dissolved in a portion of water to ensure uniform mixing. Throughout the experimental period, FHVO-NDC fully dissolved in the drinking water without visible sedimentation or particulate matter. The drinking water in both the CON and TRT groups was changes every 2 days to ensure freshness.

Mice were housed in pairs under controlled environmental conditions with a 12-h light/dark cycle and stable humidity to comply with animal welfare requirements. They were provided with standard chow pellets (WQJX Bio-technology, Wuhan, China) composed of 40% corn, 20% soybean meal, 7% fish meal, 23% wheat flour, 3% yeast, 2% dicalcium phosphate, 1.5% limestone, 0.5% NaCl, 2% soybean oil, and 1% premix. The diet contained 20.7% crude protein, 2.31% crude fiber, 4.15% crude fat, 1.24% calcium, and 0.83% phosphorus. Animals had *ad libitum* access to both feed and water, and intake was monitored throughout the study. All procedures were approved by the Animal Care and Use Committee of Hubei University of Arts and Science (No. 2025–26; Xiangyang, China) and were conducted in accordance with the ARRIVE (Animal Research: Reporting of *In Vivo* Experiments) guidelines.

### 2.2 Measurement of fatty acid components in FHVO-NDC

Fatty acid composition was analyzed using an Agilent 7,890–5,977 GC/MS system (Agilent Technologies Inc., CA, USA). The oven temperature was programmed to increase from 90 to 180 °C at a rate of 3 °C/min. Gas flow rates were set as follows: carrier nitrogen at 1 mL/min, hydrogen at 30 mL/min, and air at 400 mL/min.

### 2.3 Body condition measurements

Body length (measured from the nose to the base of the tail) and body weight were recorded every two weeks. These measurements were used to calculate body surface area and body mass index (BMI) following the equations described by Gargiulo et al. (31).


$$\text{Body surface}=0.007184\times \text{body weigh}t^{0.425}\times \text{body lengt}h^{0.725}$$

Body surface area is expressed in m^2^; body weight in kg; and body length in cm


BMI=body weightbody surface

BMI is expressed in g/m^2^; body weight in g; and body surface area in m^2^.

### 2.4 Athletic performance measurements

Following the experimental design of Jäger et al. (32) and Wei et al. (33), all mice in each group were subjected to a series of tests to assess athletic performance, including forelimb grip strength (34), exhaustive swimming test (35), wire handing test (36), and pole test (37). All behavioral assessments were performed by investigators blinded to group allocation.

#### 2.4.1 Forelimb grip strength measurement

Grip strength was assessed using a force transducer (Yuyan Instruments, Shanghai, China) equipped with a metal mesh. Measurements were conducted on days 1, 14, 28, 42, 56, and 70. The maximum force recorded (in grams) was considered the measure of absolute grip strength (38).

#### 2.4.2 Exhaustive swimming test

On day 65, each mouse was fitted with a lead sheet equivalent to 5% of its body weight and placed in a cylindrical pool (65 cm height, 20 cm diameter) filled with 40 cm of water maintained at 27 ± 1 °C (38). Endurance was defined as the time to exhaustion, characterized by the loss of coordinated movements or failure to surface within 7 s. Floating, struggling, and necessary swimming movements were included in the time until exhaustion or potential drowning (39). The total duration (seconds) was recorded as the endurance score.

#### 2.4.3 Wire hanging test

On day 67, mice were tested using a metal mesh bordered with duct tape. A lead sheet weighing 30% of each mouse’s body weight was attached to the tail. After the mouse was placed on the mesh and allowed to grip, the mesh was gently shaken and then inverted 20 cm above soft bedding. The time to fall was recorded (40), and the total duration (seconds) served as the performance score.

#### 2.4.4 Pole test

On day 68, each mouse was placed on top of a 60 cm vertical pole (1 cm diameter) with a cardboard cap to prevent upward climbing. The pole was fixed on a triangular base and positioned inside the cage to encourage descend. Timing began when the mouse was stationary on the pole, and both the time to turn completely downward and the total time to reach the floor were recorded (41). Each mouse completed three trials. Mice that fell immediately were assigned the minimum score of 0 s. A maximum score of 1,000 s was set, and the final score was calculated by subtracting the recorded time from 1,000, ensuring that mice with better performance received higher scores.

### 2.5 Sample collection

On day 70, mice were anesthetized with 1% pentobarbital, and blood samples were collected via orbital extraction. Mice were then euthanized by cervical dislocation. Blood samples were kept at 4 °C for 30 min and centrifuged at 3,000 rpm for 15 min. The resulting serum was transferred to clean centrifuge tubes and stored at −80 °C. Due to technical issues during blood collection and handling errors during sample processing, two samples from the TRT group were lost and one sample yielded an insufficient serum volume for metabolomic analysis. Therefore, to ensure balanced sample numbers across groups, five serum samples per group were used for subsequent metabolomic profiling.

The gastrocnemius muscles from both hind limbs were harvested. The left gastrocnemius was homogenized in lysis buffer (20 mM Tris, 1 mM ethylenediaminetetraacetic acid, 100 mM sodium chloride, and 0.5% (v/v) Triton X-100) and centrifuged at 3,000 rpm at 4 °C for 10 min to obtain the supernatant. The right gastrocnemius was fixed in 4% paraformaldehyde for paraffin embedding.

Colon contents were collected, placed in sterile bags, and stored at −20 °C until DNA extraction.

Due to funding constraints in the early stage of this project, six samples of gastrocnemius muscles and colon contents were therefore randomly selected from each group for subsequent ELISA assays, myofiber diameter analysis, and intestinal microbiota analysis.

### 2.6 Relative weight of gastrocnemius

The relative weight of the gastrocnemius was calculated using the following equation:


Tissue index=Tissue weightBody weight

Tissue index is expressed in %; tissue weight in g; body weight in g.

### 2.7 ELISA assays

Supernatants from homogenized gastrocnemius tissues were used to determine the levels of glycogen, pyruvate kinase, succinate dehydrogenase, Na^+^/K^+^-ATPase, and Ca^2+^-Mg^2+^-ATPase. All analyses were performed using commercial ELISA kits according to the manufacturer’s instructions (Jingmei Biotechnology, Jiangsu, China).

### 2.8 Gastrocnemius histology analysis

Paraformaldehyde-fixed gastrocnemius samples were dehydrated in a graded ethanol series (70, 80, 90, 95, and 100%) followed by xylene. Samples were embedded in paraffin and kept in an oven at 60 °C. After 12 h, samples were removed, placed into histological cassettes, transferred to paper molds, and covered with molten paraffin. Once solidified, paraffin blocks were stored under refrigeration until sectioning (42).

Serial sections (3 μm thickness) were cut perpendicular to the orientation of the myofibers using a cryostat. Paraffin ribbons were mounted onto coated glass slides and dried at 60 °C for 2 h to remove residual paraffin. Slides were then deparaffinized and rehydrated with xylene and graded ethanol solutions. Hematoxylin and eosin staining was performed following standard protocols (42).

After staining, samples were dehydrated again, mounted, and examined under a light microscope equipped with a ScopePhoto system (LY-WN 300, Hangzhou Scopetek Opto-Electric Co., Ltd.). A minimum of 150 intact and appropriately oriented muscle fibers were measured across five fields of view using a 40 × objective lens. Assuming fibers were circular, cross-sectional area was converted to diameter using the formula:


Diameter=2Muscle fiber cross_sectional areaπ

Diameter is expressed in μm; muscle fiber cross-sectional area in μm^2^.

The mean value was calculated to represent the average myofiber diameter (43).

### 2.9 Serum metabolome analysis

Five serum samples were selected from each group for metabolomic analysis, consistent with the sample sizes used in previous studies (44, 45). For each sample, 400 μL of a methanol-acetonitrile solution (1,1, v/v) was added to 100 μL of serum, followed by vortexing for 30 s. The mixture was then sonicated in an ice bath for 10 min and incubated at −40 °C for 1 h. Subsequently, 400 μL of the supernatant was transferred to a protein precipitation plate and shaken for 2 min at 6 psi.

Chromatographic separation was performed using a Vanquish ultra-high-performance liquid chromatography system (Thermo Fisher Scientific) equipped with a Phenomenex Kinetex C18 column (2.1 mm × 50 mm, 2.6 μm). The mobile phase consisted of water containing 0.01% acetic acid (phase A) and a 1:1 (v/v) mixture of isopropanol and acetonitrile (phase B). The sample tray was maintained at 4 °C and the injection volume was 2 μL.

Mass spectrometric detection was conducted using an Orbitrap Exploris 120 mass spectrometer controlled by Xcalibur software (version 4.4). The major parameters were as follows: sheath gas flow rate, 50 Arb; auxiliary gas flow rate, 15 Arb; capillary temperature, 320 °C; full MS resolution, 60,000; MS/MS resolution, 15,000; normalized collision energy, 20/30/40; spray voltage of 3.8 kV (positive mode) and −3.4 kV (negative mode). Raw data files were converted to mzXML format using ProteoWizard for subsequent metabolite identification.

After conversion, the raw data were further processed to ensure analytical robustness. Peaks were filtered to remove noise, and variables were screened using the relative standard deviation to exclude unstable features. Peaks were retained only if the proportion of missing values was ≤50% across samples. Missing values were imputed using a half-minimum approach. Data normalization was then performed using an internal standard to correct for technical variation. All identified metabolites were subsequently mapped to KEGG databases.

### 2.10 Intestinal microbiota analysis

Intestinal microbiota analysis was performed using six colon content samples from each group, consistent with the sample size used in previous study (46, 47). Total DNA was extracted using a magnetic Soil and Stool DNA Kit (cat# DP712, TIANGEN Biotech Co., Ltd., Beijing, China). DNA concentration and purity were assessed using a Nanodrop One (Thermo Fisher Scientific, MA, USA) and 1% agarose gel electrophoresis. Extracted DNA was diluted to 1 ng/μL with sterile water and stored at −20 °C.

The V3-V4 hypervariable regions of the bacterial 16S rRNA gene were amplified using universal primers. PCR products were purified with a Qiagen Gel Extraction Kit (cat# 28706, Qiagen, Germany), and DNA quality was further evaluated using a Qubit 2.0 dsDNA HS Assay Kit (cat# Q32854, Invitrogen). After concentration normalization using GeneTools Analysis Software (Version 4.03.05.0, SynGene), PCR products were pooled according to equal-mass principles. The pooled products were purified with an E. Z. N. A.^®^ Gel Extraction Kit (Omega, USA) and eluted in TE buffer. Library construction was performed following the ALFA-SEQ DNA Library Prep Kit protocol. Library fragment size and concentration were assessed using a Qsep400 system (Hangzhou Houze Biotechnology Co., Ltd., China) and a Qubit 4.0 fluorometer (Thermo Fisher Scientific, Waltham, USA), respectively. Amplicon libraries were sequenced using paired-end 250 bp mode on either an Illumina MiSeq or an MGI DNBSEQ platform (Guangdong Magigene Biotechnology Co., Ltd., Guangzhou, China). The average sequencing depth was approximately 50,000–80,000 raw reads per sample, a range comparable to previously published V3-V4 16S studies (48, 49).

Paired-end raw reads were quality-filtered using fastp (version 0.14.1) with sliding-window trimming, and primers were removed using cutadapt. High-quality paired-end reads were merged using USEARCH (V10), and non-compliant or chimeric sequences were removed to obtain clean tags. High-quality sequences were clustered into operational taxonomic units (OTUs) at 97% sequence identity using Uparse v7.0.1001. We selected OUT-based clustering to maintain consistency with previous studies in this research field and with the historical datasets used for comparison (50). Taxonomic assignment was performed in QIIME v1.9.1 using the SILVA database with a confidence threshold of 0.8. Sequences annotated as chloroplasts or mitochondria were excluded.

Rarefaction curves were generated using R software (V5.1.3) to evaluate sequencing depth. All samples were subsequently rarefied to an even depth of 20,000 reads to ensure consistent alpha and beta diversity comparisons, as rarefaction curves plateaued near this depth. Alpha diversity indices and beta diversity analyses were performed in R (v5.1.3). Differentially abundant taxa were identified using Linear discriminant analysis Effect Size (LEfSe), applying the standard workflow (Kruskal-Wallis test, Wilcoxon post-hoc test, and LDA). An LDA threshold of 2.0 was used to identify taxa with meaningful effect sizes. To reduce potential false-positive findings, LEfSe results were cross-checked with conventional statistical tests. A Random Forest classifier was used to identify key taxa associated with group differences. Model performance was evaluated using 10-fold cross-validation with 5 repeated runs, and model robustness was further assessed using permutation importance. Hyperparameters were optimized using grid search. These validation steps ensured stable model performance and reduced overfitting. Final valid tags and comprehensive OTU tables were compiled for downstream evaluation of species richness and community composition using R (v5.1.3).

### 2.11 Statistical analysis

The normality of all data was assessed using the PROC UNIVARIATE procedure in SAS software (version 9.4, 2014, SAS Inst. Inc., Cary, NC, USA). Results are presented as means ± pooled standard error of the mean, with statistical significance defined as *p* < 0.05.

Body condition parameters, feeding behavior, athletic performance, muscle fatigue-related biochemical indicators, gastrocnemius index, and myofiber diameter were analyzed using One-way ANOVA.

For metabolomics, Orthogonal Projections to Latent Structures-Discriminant Analysis (OPLS-DA) was used to identify metabolic differences between groups. Potential biomarkers were screened based on Variable Importance in Projection (VIP) values from OPLS-DA and *p*-values from Student’s *t*-tests. Metabolites with VIP > 1 and *p* < 0.05 were considered significant.

For intestinal microbiota analysis, alpha diversity indices (Chao1, Simpson, Shannon, Pielou) were calculated, and between-group differences were assessed using One-way ANOVA. Beta diversity significance testing was performed using the vegan and pegas packages in R, including Anosim, MRPP, Adonis, and Amova. LEfSe analysis was conducted using normalized species abundance to identify differentially enriched taxa. Random Forest analysis was applied to construct predictive models based on OTU abundance, identify key taxa, and validate results using a custom validation set.

Spearman’s correlation analysis was conducted to explore associations between key bacteria and selected serum metabolites.

## 3 Results

Fatty acid composition analysis of FHVO-NDC in the present study revealed that this product contained high levels of saturated fatty acids (37.44%), ranked as follows: lauric acid (14.26%), stearic acid (7.39%), myristic acid (5.37%), palmitic acid (4.42%), capric acid (2.67%), caprylic acid (2.25%), arachidic acid (0.96%), and behenic acid (0.12%). In contrast, unsaturated fatty acids were relatively less abundant, including oleic acid (3.28%), linoleic acid (0.34%), and eicosenoic acid (0.19%) (Table 1).

**Table 1:** Composition of fatty acid components in fully hydrogenated vegetable oil-based non-dairy creamer, %.

Lauric acid (C12:0)	14.26
Myristic acid (C14:0)	5.37
Palmitic acid (C16:0)	4.42
Stearic acid (C18:0)	7.39
Oleic acid (C18:1)	3.28
Linoleic acid (C18:2)	0.34
Arachidic acid (C20:0)	0.96
Eicosenoic acid (C20:1)	0.19
Behenic acid (C22:0)	0.12
Caprylic acid (C8:0)	2.25
Capric acid (C10:0)	2.67
Saturated fatty acids	37.44
Unsaturated fatty acids	3.81
Ratios for saturated fatty acids and unsaturated fatty acids	9.83

Changes in the body condition of mice following FHVO-NDC intake are shown in Table 2. No significant differences in body weight, body length, body surface area, or BMI were observed at any measurement timepoint.

**Table 2:** Changes in body condition of mice following intake of fully hydrogenated vegetable oil-based non-dairy creamer (FHVO-NDC; *n* = 8).

Items	CON	TRT	SEM	*P*-value
Body weight (g)				
Day 1	22.183	22.190	7.222	0.998
Day 14	24.146	23.493	8.445	0.887
Day 28	24.370	23.936	5.904	0.893
Day 42	24.886	25.914	5.601	0.735
Day 56	26.414	26.505	4.674	0.971
Day 70	26.270	26.178	4.631	0.971
Body length (cm)				
Day 1	8.238	8.238	0.951	1.000
Day 14	8.700	8.663	0.772	0.929
Day 28	8.800	8.825	0.723	0.949
Day 42	8.800	8.900	0.725	0.800
Day 56	8.950	9.063	0.627	0.741
Day 70	8.975	9.138	0.604	0.619
Body surface (cm^2^)				
Day 1	65.317	65.574	14.365	0.974
Day 14	70.586	68.934	14.548	0.835
Day 28	71.531	71.152	11.344	0.951
Day 42	72.188	74.021	10.769	0.754
Day 56	75.085	75.772	9.208	0.891
Day 70	74.974	75.903	8.920	0.848
BMI (g/cm^2^)				
Day 1	0.328	0.334	0.038	0.778
Day 14	0.337	0.327	0.051	0.722
Day 28	0.338	0.331	0.030	0.695
Day 42	0.342	0.346	0.032	0.792
Day 56	0.350	0.347	0.023	0.829
Day 70	0.348	0.343	0.023	0.685

SEM, standard error of the mean; BMI, body mass index. Data were analyzed using one-way ANOVA. Mice in the CON group received regular water. Mice in the TRT group received water supplemented with FHVO-NDC at a concentration of 0.167%.

Additionally, FHVO-NDC intake had no significant effects on appetite-related parameters, including daily feed intake and water consumption (Table 3).

**Table 3:** Changes in appetite of mice following intake of fully hydrogenated vegetable oil-based non-dairy creamer (FHVO-NDC; *n* = 4).

Items	CON	TRT	SEM	*P*-value
Feed intake (g)				
Days 1–14	7.495	7.590	4.424	0.980
Days 15–28	8.340	7.918	4.837	0.918
Days 29–42	8.230	7.974	4.825	0.950
Days 43–56	8.765	8.461	1.325	0.786
Days 57–70	8.901	8.766	2.555	0.951
Water intake (mL)				
Days 1–14	12.092	19.385	10.849	0.416
Days 15–28	11.946	13.863	9.150	0.805
Days 29–42	15.970	19.039	4.568	0.416
Days 43–56	9.244	8.872	3.873	0.910
Days 57–70	10.208	11.424	1.454	0.302

SEM, standard error of the mean. Data were analyzed using one-way ANOVA. Mice in the CON group received regular water. Mice in the TRT group received water supplemented with FHVO-NDC at a concentration of 0.167%.

Consumption of FHVO-NDC significantly reduced forelimb grip strength on day 42 (*p* = 0.001), 56 (*p* = 0.006), and 70 (*p* < 0.001) (Table 4). Moreover, mice in the TRT group exhibited poorer performance in the exhaustive swimming test (*p* = 0.030), wire hanging test (*p* = 0.012), and pole test (*p* = 0.011) (Table 4).

**Table 4:** Changes in forelimb grip strength and athletic performance of mice following intake of fully hydrogenated vegetable oil-based non-dairy creamer (FHVO-NDC; *n* = 8).

Items	CON	TRT	SEM	*P*-value
Forelimb grip strength (g)				
Day 1	36.768	35.103	12.691	0.809
Day 14	44.359	39.928	8.193	0.311
Day 28	47.743	41.784	9.141	0.218
Day 42	47.641	35.889	7.796	0.001
Day 56	48.153	36.514	8.919	0.006
Day 70	52.314	35.195	10.203	<0.001
Score for the athletic performance				
Exhaustive swimming test	752.250	405.250	320.222	0.030
Wire hanging test	23.059	6.336	11.970	0.012
Pole test	860.222	370.611	349.815	0.011

SEM, standard error of the mean. Data were analyzed using one-way ANOVA. Mice in the CON group received regular water. Mice in the TRT group received water supplemented with FHVO-NDC at a concentration of 0.167%.

The relative weight of the gastrocnemius muscle was significantly reduced by FHVO-NDC administration (*p* = 0.021; Table 5). In addition, FHVO-NDC consumption resulted in decreased myofiber diameter (*p* < 0.001), reduced muscle glycogen storage (*p* < 0.001), and lower levels of key metabolic and ionic transport enzymes, including pyruvate kinase (*p* < 0.001), succinate dehydrogenase (*p* < 0.001), Na^+^/K^+^-ATPase (*p* < 0.001), and Ca^2+^-Mg^2+^-ATPase (*p* = 0.001) (Table 5).

**Table 5:** Changes in muscle traits, muscle glycogen levels, and the activities of enzymes related to muscle fatigue of mice following intake of fully hydrogenated vegetable oil-based non-dairy creamer (FHVO-NDC).

Items	CON	TRT	SEM	*P*-value
The relative weight of gastrocnemius muscle (%, *n* = 8)	0.553	0.385	0.129	0.021
Diameter of myofiber (μm, *n* = 6)	26.440	18.247	4.625	<0.001
Muscle glycogen levelsv (mg/L, *n* = 6)	6.473	5.878	0.549	<0.001
Pyruvate kinase (ng/L, *n* = 6)	94.658	83.145	10.549	<0.001
Succinate dehydrogenase (μmol/L, *n* = 6)	37.406	33.505	2.887	<0.001
Na^+^/K^+^-ATPase (pg/mL, *n* = 6)	62.394	54.575	7.162	<0.001
Ca^2+^-Mg^2+^-ATPase (ng/L, *n* = 6)	35.355	30.763	4.712	0.001

SEM, standard error of the mean. Data were analyzed using one-way ANOVA. Mice in the CON group received regular water. Mice in the TRT group received water supplemented with FHVO-NDC at a concentration of 0.167%.

**Figure 2:**
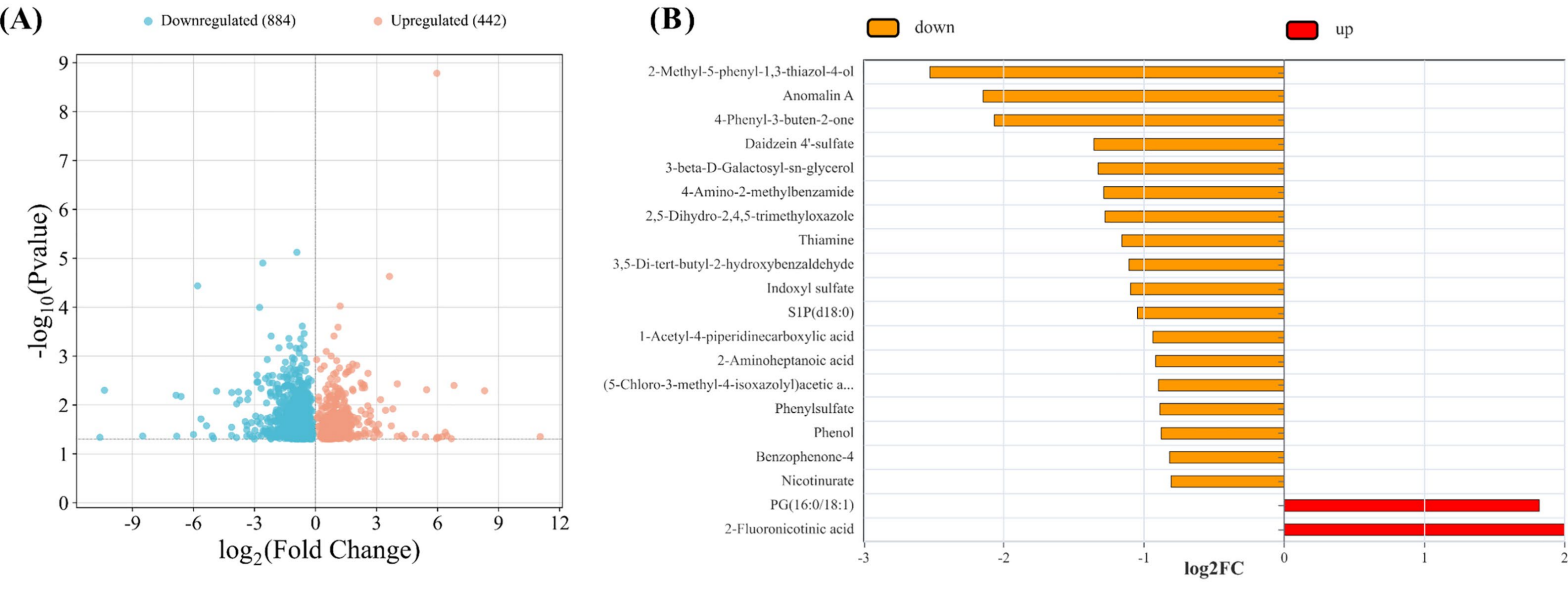
Effects of fully hydrogenated vegetable oil-based non-dairy creamer intake on serum metabolite profiles in mice (*n* = 5). The volcano plot shows the differential serum metabolites between groups **(A)**. The bar chart presents the top 20 metabolites ranked by fold change **(B)**.

Serum metabolite profiling revealed widespread metabolic alterations following FHVO-NDC intake. A total of 442 metabolites were significantly upregulated and 884 were downregulated (Figure 2A). Among these, 2-fluoronicotinic acid and PG (16:0/18:1) were identified as key upregulated metabolites based on their high rankings in the top 20 metabolites by log_2_ fold change (Figure 2B). Comparison analysis further showed that levels of 2-fluoronicotinic acid (*p* = 0.022) and PG (16:0/18:1) (*p* = 0.040) were significantly higher in the TRT group than in the CON group (Table 6).

**Table 6:** Effects of fully hydrogenated vegetable oil-based non-dairy creamer (FHVO-NDC) intake on intestinal *Alistipes* abundance, serum 2-Fluoronicotinic acid and PG (16.0/18.1) levels, as well as their Spearman correlation relationship.

Items	CON	TRT	SEM	*P*-value
*Alistipes*	0.005	0.013	0.005	0.007
2-Fluoronicotinic acid	6.061	7.042	0.766	0.022
PG(16:0/18:1)	6.813	7.341	0.421	0.040
Variables	2-Fluoronicotinic acid	PG(16:0/18:1)		
*Alistipes*	0.685*	0.248		

SEM, standard error of the mean. ^*^p < 0.05. Data were analyzed using one-way ANOVA. Mice in the CON group received regular water. Mice in the TRT group received water supplemented with FHVO-NDC at a concentration of 0.167%.

Regarding the intestinal microbiota, alpha diversity indexes, including Chao1, Simpson, Shannon, and Pielou did not differ significantly between groups (Table 7). Similarly, beta diversity analyses using Adonis, Amova, Anosim, and MRPP showed no statistically significant separation among microbial communities (Table 8).

**Table 7:** Changes in alpha diversity of mice intestinal microbiota following intake of fully hydrogenated vegetable oil-based non-dairy creamer (FHVO-NDC; *n* = 6).

Items	CON	TRT	SEM	*P*-value
Chao1	1163.500	1127.000	158.889	0.722
Pielou	0.643	0.606	0.050	0.249
Simpson	0.055	0.056	0.029	0.942
Shannon	4.535	4.258	0.429	0.307

SEM, standard error of the mean. Data were analyzed using one-way ANOVA. Mice in the CON group received regular water. Mice in the TRT group received water supplemented with FHVO-NDC at a concentration of 0.167%.

**Table 8:** The *P*-value of beta diversity in intestinal microbiota of mice as affected by fully hydrogenated vegetable oil-based non-dairy creamer intake (*n* = 6).

Adonis	Amova	Anosim	MRPP
0.121	0.111	0.154	0.136

**Figure 3:**
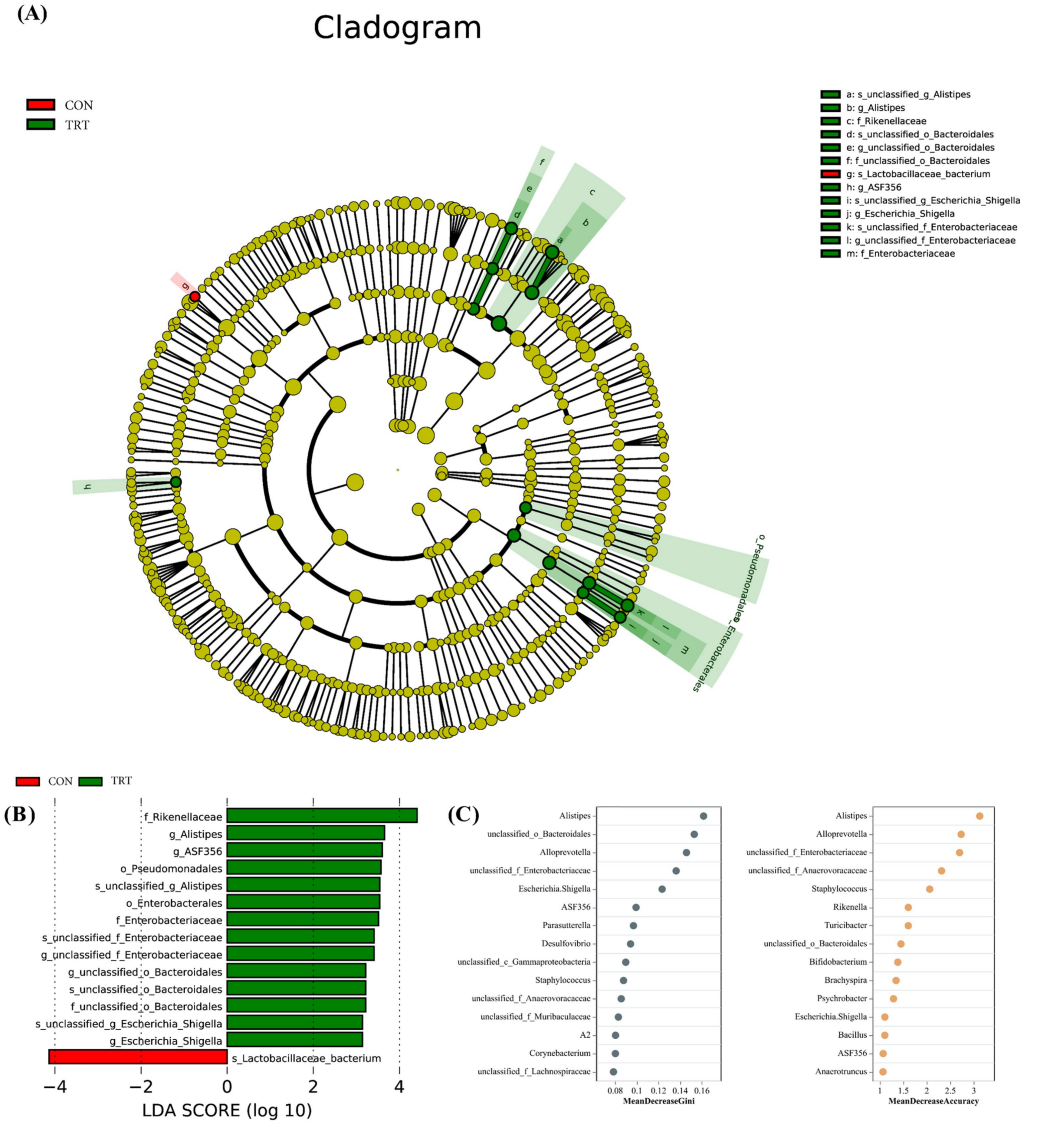
Effects of fully hydrogenated vegetable oil-based non-dairy creamer intake on intestinal microbiota in mice (*n* = 6). The cladogram **(A)** and linear discriminant analysis effect size analysis **(B)** indicate differences in the phylogenetic distribution of lineages among groups. The random forest ensemble learning method identifies key signature taxa of the intestinal microbiota based on mean decrease accuracy and mean decrease Gini indicators **(C)**.

LEfSe analysis identified several taxa that differed significantly between groups. *Alistipes*, *ASF356* (*Clostridium*), and *Escherichia_Shigella* were enriched in mice consuming FHVO-NDC, whereas *Lactobacillus* was the signature genus in the CON group (Figures 3A,B). Random forest analysis further evaluated the relative importance of these taxa in relation to muscle-related phenotypes. Both the mean decrease accuracy and mean decrease Gini indicators ranked *Alistipes* as the most influential genus. Comparison analysis confirmed that *Alistipes* abundance was significantly higher in the TRT group than in the CON group (*p* = 0.007; Table 6).

Furthermore, Spearman correlation analysis revealed that the abundance of *Alistipes* was positively correlated with serum 2-fluoronicotinic acid levels (*p* = 0.029; Table 6).

## 4 Discussion

Body condition and feeding behavior are critical parameters for evaluating the general health status of experimental animals, as they reflect both physiological stability and overall adaptability to experimental conditions. Under adverse or stressful conditions, animals often display poorer body condition, abnormal feeding patterns, or reduced food intake, which may indicate discomfort or systemic dysfunction (51). Ali et al. (52) reported that the body weight of mice consuming non-dairy creamer remained comparable to that of mice consuming milk, suggesting that the intake of such products does not necessarily disturb basic physiological functions. Consistent with these findings, the present study showed that consumption of water containing FHVO-NDC did not affect any parameters related to body condition or feeding behavior. These observations collectively suggest that FHVO-NDC intake did not induce acute physical discomfort in mice and that any subsequent physiological or metabolic changes identified in this study are unlikely to be attributed to poor feeding behavior or compromised body condition.

From the perspective of its fatty acid composition, saturated fatty acids are the primary components of FHVO-NDC. High intake of saturated fatty acids has been shown to increase the risk of metabolic syndrome (7, 8) and to promote chronic low-grade inflammation (9). Both metabolic syndrome and chronic inflammation are recognized risk factors for impaired musculoskeletal health, and they have been associated with reductions in muscle mass and strength (10, 11). Moreover, chronic inflammation can directly disrupt muscle metabolism, impair neuromuscular function, and ultimately reduce athletic performance (12). Therefore, strategies that prevent metabolic syndrome or suppress chronic inflammation have been shown to effectively improve physical performance and muscle function (53, 54). Although one of the saturated fatty acids in FHVO-NDC, lauric acid, has been reported to support myofiber formation and promote skeletal muscle growth (55, 56), other major saturated fatty acids present in FHVO-NDC exert detrimental effects. For example, stearic acid promotes proinflammatory cytokine release (57), contributes to cell growth inhibition, and induces cellular inflammation (58). Myristic acid contributes to adipose tissue inflammation (59) and has been linked to hypercholesterolemia (60). Palmitic acid, the most widely studied saturated fatty acid, is known to trigger metabolic inflammation, mitochondrial fragmentation, and reductions in mitochondrial membrane potential, all of which may impair exercise capacity and energy production (61, 62). Additionally, myristic acid has been shown to aggravate palmitic acid-induced inflammation (63), suggesting a synergistic inflammatory effect when multiple saturated fatty acids coexist. Taken together, these findings suggest that although FHVO-NDC contains some beneficial components, its overall saturated fatty acids components may contribute to reduced athletic performance.

As expected, throughout the experiment, mice in the TRT group showed insufficient strength to complete the swimming, wire-hanging, and pole-climbing tasks for extended periods. Athletic performance is closely linked to skeletal muscle integrity, particularly muscle mass and myofiber diameter (64, 65). Histologically, skeletal muscle is composed of long, cylindrical myofibers arranged in bundles, and these myofibers serve as the fundamental functional units of muscle tissue (66). Numerous studies have shown that myofiber diameter is directly proportional to muscle strength and total muscle mass (67–69). Conversely, a reduction in myofiber diameter is considered a hallmark histopathological feature of skeletal muscle atrophy (70). Therefore, larger myofibers and greater muscle mass generally correspond to enhanced athletic capacity (71, 72). In the present study, the significant reductions observed in gastrocnemius muscle mass and myofiber diameter in the TRT group suggest that FHVO-NDC intake may induce muscle atrophy in mice, which is consistent with their impaired athletic performance.

Furthermore, muscle glycogen content, a major energy reserve within muscle tissue, is typically positively correlated with muscle mass. Glycogen represents the primary storage form of carbohydrates in animals (73). During exercise, glycogen is degraded into glucose, which subsequently undergoes metabolic processes to generate ATP (74). Larger muscles generally possess a greater capacity for glycogen storage, allowing for more sustained energy output during physical activity (75). Insufficient glycogen storage leads to rapid fatigue and impaired athletic performance (76), and several studies have confirmed that interventions promoting glycogen synthesis enhance exercise capacity (77). Therefore, the reductions in glycogen storage capacity may also contribute to the decline in athletic performance. In addition to glycogen, muscle energy metabolism depends on the activity of several key enzymes involved in ATP production. We evaluated the levels of pyruvate kinase, succinate dehydrogenase, Na^+^/K^+^-ATPase, and Ca^2+^-Mg^2+^-ATPase, enzymes essential for glycolysis, the tricarboxylic acid cycle, and ion transport-dependent ATP consumption (78, 79). Reduced levels of these enzymes has been linked to impaired muscle energy metabolism and decreased exercise capacity, whereas higher levels support improved muscle function and endurance (78, 79). In this study, FHVO-NDC intake decreased all four ATP production-related enzymes, indicating substantial disruption of muscle energy metabolism. Therefore, the combined reduction in ATP-producing enzymes and lower glycogen concentrations directly compromises the energy supply available for muscle contraction, providing a mechanistic explanation for the observed decline in athletic performance.

Circulating metabolites can regulate the levels of enzymes involved in cellular energy production through multiple biochemical mechanisms (80). In the present study, 2-fluoronicotinic acid and PG(16:0/18:1) were identified as the two key upregulated metabolites, as both were ranked within the top 20 metabolites based on log_2_ fold change values. PG(16:0/18:1), a phosphatidylglycerol species, has been previously associated with reductions in skeletal muscle mass and functional capacity (81). Consistently, Hinkley et al. (81) demonstrated that elevated phosphatidylglycerol levels are characteristic of sarcopenic muscle and show a negative correlation with muscle volume, suggesting a potential role in muscle wasting. In addition, 2-fluoronicotinic acid has been reported to inhibit nicotinate phosphoribosyltransferase (NaPRTase) activity in human platelet lysate (82). NaPRTase catalyzes the synthesis of nicotinic acid mononucleotide, the direct precursor required for the formation of nicotinamide adenine dinucleotide (83). As nicotinamide adenine dinucleotide availability is closely linked to efficient ATP generation through oxidative metabolism (84), suppression of this pathway may impair cellular energy production. Therefore, the elevated levels of PG (16:0/18:1) and 2-fluoronicotinic acid observed in this study likely contribute to the reduced muscle strength and diminished ATP production capacity.

Circulating metabolites are strongly influenced by the intestinal microbiota (85). In this study, we first evaluated the overall structural characteristics of the intestinal microbiota by assessing alpha diversity indices, including Chao1, Simpson, Shannon, and Pielou, which reflect microbial richness, diversity, stability, and evenness, respectively (86). To further examine compositional differences among groups, beta diversity was analyzed using multiple statistical approaches, including Adonis, Amova, Anosim, and MRPP, thereby providing a robust assessment of community structural variation. Results from both alpha diversity and beta diversity analyses showed no significant differences among groups, suggesting that FHVO-NDC intake did not alter the richness, diversity, stability, or evenness of the gut microbial community. However, it is important to note that unchanged alpha diversity and beta diversity metrics do not necessarily reflect functional or taxonomic stability. Microbial communities can maintain comparable richness and overall evenness while undergoing taxonomic turnover or shifts in specific bacterial populations (86, 87). Therefore, despite the absence of diversity changes, FHVO-NDC consumption may still influence particular microbial taxa that contribute to metabolic alterations and the observed decline in athletic performance.

To further investigate microbial differences, we employed the LEfSe algorithm to identify signature taxa within the intestinal microbiota. The analysis revealed that *Alistipes*, *ASF356* (*Clostridium*), and *Escherichia_Shigella* were signature genera enriched in mice consuming FHVO-NDC, whereas *Lactobacillus* was identified as the key genus in the CON group. The *Lactobacillus* genus is widely recognized as beneficial and has been associated with improved muscle function, metabolic stability, and enhanced exercise capacity (88). In contrast, *Clostridium*, *Alistipes*, and *Escherichia_Shigella* are generally regarded as harmful bacteria that have been linked to inflammation, metabolic dysfunction, and impaired muscle performance (89–91). These findings suggest that FHVO-NDC intake may shift the intestinal microbiota toward a more deleterious composition despite the absence of changes in overall microbial diversity.

To further evaluate the relative contributions of these genera, we applied a random forest ensemble learning method, which provides an importance ranking based on mean decrease accuracy and mean decrease Gini. Both indicators consistently ranked *Alistipes* as the most influential genus. This observation strongly supports the notion that the expansion of *Alistipes* plays a central role in mediating the negative effects of FHVO-NDC on muscle function and athletic performance. Similar conclusions were reached by Lee et al. (90), who identified *Alistipes* as a predominant genus in sarcopenic and aged mice. Furthermore, Kim et al. (92) reported a positive correlation between *Alistipes* abundance and chronic fatigue syndrome. Together, these studies reinforce the concept that *Alistipes* is closely associated with muscle weakness phenotypes. Mechanistically, *Alistipes* possesses the genes required for fatty acid degradation and can efficiently utilize dietary fatty acids as a growth substrate (93). Given the saturated fatty acid-rich composition of FHVO-NDC, the increased abundance of *Alistipes* in the TRT group is therefore biologically plausible. Moreover, Spearman’s correlation analysis further demonstrated that 2-fluoronicotinic acid was positively correlated with *Alistipes* abundance, suggesting a potential interaction between circulating metabolite production and microbial community shifts. This correlation indicates that increases in *Alistipes* may contribute to metabolic alterations.

Therefore, this study provides an integrated assessment of the effects of FHVO-NDC intake on athletic performance by combining behavioral tests, muscle histology, biochemical analysis, metabolomics, and gut microbiota profiling. The identification of key metabolites [2-fluoronicotinic acid and PG(16:0/18:1)] and harmful bacteria such as *Alistipes* offers new mechanistic insights into how hydrogenated fat-based creamers may impair muscle function.

However, several limitations should be noted. First, the study was conducted only in mice, and their physiological responses may not fully represent humans. Second, although associations between metabolites, microbes, and muscle performance were observed, causal relationships were not directly validated using functional experiments. Third, only one dose and duration were tested; dose–response or time-dependent effects remain unknown. Fourth, the relatively small sample size may reduce statistical power. In addition, underlying molecular pathways such as mitochondrial function and muscle-fiber remodeling require further clarification.

Despite these limitations, the findings suggest that FHVO-NDC intake may negatively affect muscle traits and athletic performance by altering serum metabolism, reducing glycogen storage, decreasing ATP-producing enzymes, and increasing harmful gut bacteria. These results raise concerns about frequent consumption of hydrogenated fat-based creamers. Although direct dietary recommendations for athletes cannot yet be made, limiting excessive intake may be prudent.

Future studies should include dose–response experiments, sex-difference analyses, and human clinical validation. Mechanistic research on mitochondrial bioenergetics and metabolite-microbiota interactions will also help translate these findings into evidence-based dietary guidance for physically active individuals.

## 5 Conclusion

Based on the findings of this study, although no changes in body condition or feeding behavior were observed, the consumption of FHVO-NDC resulted in significant negative effects on athletic performance of mice. This decline is closely associated with decreased muscle glycogen storage and reduced levels of enzymes related to muscle fatigue, driven by reductions in muscle mass and myofiber diameters, both of which are crucial for energy production during exercise. Furthermore, serum metabolomics and intestinal microbiota analyses revealed that FHVO-NDC intake promoted the accumulation of metabolites, such as PG (16:0/18:1) and 2-fluoronicotinic acid, which are associated with impaired muscle function and reduced ATP production. Alterations in the intestinal microbiota, particularly the increase in *Alistipes*, *Escherichia_Shigella*, and *Clostridium*, further contributed to the observed decline in athletic performance.

Therefore, our findings provide preliminary evidence that hydrogenated fat-based creamers could detrimentally alter metabolic and microbiome profiles relevant to exercise performance, and may further negatively affect muscle traits, leading to reduced glycogen stores and decreased levels of ATP-producing enzymes, ultimately compromising athletic performance of mice.

## Data Availability

The datasets presented in this study can be found in online repositories. The names of the repository/repositories and accession number(s) can be found at: https://figshare.com/, https://figshare.com/articles/dataset/_/27245835, https://figshare.com/articles/dataset/_/27245832.
